# Carrier Effect in Development of Rifampin Loaded Proliposome for Pulmonary Delivery: A Quality by Design Study

**DOI:** 10.34172/apb.2022.032

**Published:** 2021-05-18

**Authors:** Elahehnaz Parhizkar, Delaram Sadeghinia, Hamed Hamishehkar, Shadi Yaqoubi, Ali Nokhodchi, Shohreh Alipour

**Affiliations:** ^1^Department of Pharmaceutics, School of Pharmacy, Shiraz University of Medical Sciences, Shiraz, Iran.; ^2^Drug Applied Research Center, Tabriz University of Medical Sciences, Tabriz, Iran.; ^3^Biotechnology Research Center, Student Research Committee and Faculty of Pharmacy, Tabriz University of Medical Sciences, Tabriz, Iran.; ^4^Pharmaceutics Research Laboratory, School of Life Sciences, University of Sussex, Brighton, BN1 9QJ, UK. 5 Pharmaceutical Sciences Research Center and Department of Quality Control, School of Pharmacy, Shiraz University of Medical Sciences, Shiraz, Iran.; ^5^Pharmaceutical Sciences Research Center and Department of Quality Control, School of Pharmacy, Shiraz University of Medical Sciences, Shiraz, Iran.

**Keywords:** aerosolization properties, carrier porosity, dry powder, proliposome, rifampin

## Abstract

***Purpose:*** Pulmonary tuberculosis (TB) is a worldwide life-threatening infection. Therecommended anti-TB regimen contains oral administration of classical first-line drugs suchas rifampin for 6-24 months which often leads to low patient compliance due to high adverseeffects; therefore, lung localized pulmonary delivery of anti-TB agents may be a suitablealternative. Proliposomes free-flowing powders are well-known carriers for lung delivery sincethey can form liposomes by hydration. Liposomes are safe and useful carriers for lung deliverydue to their phospholipid structure.

***
Methods:*** Porous lactose and mannitol as proliposome carriers were prepared by spray dryingtechnique using sucrose and citric acid as templating agents. Design Expert® software wasused to develop forty formulations based on the porous and non-porous carriers, which werecharacterized with respect to their weight yield, density, and flowability. Rifampin-loadedhydrated liposomes were produced and evaluated for size, morphology, loading capacityand encapsulation efficiency. The optimized proliposomes *in vitro* release and aerosolizationproperties were evaluated. Solid-state analysis was confirmed by differential scanningcalorimetry (DSC).

***
Results:*** Porous lactose surface area was 80 folds higher than non-porous one, respectively.Optimized porous-based proliposome indicated the acceptable aerosolization properties,including mass median aerodynamic diameter (MMAD) of 6.21 ± 0.36 μm and fine particlefraction (FPF) of 9.17 ± 0.18% with a fast rifampin release (80%) within one hour. DSC resultsproved that there was no change in the solid-state of rifampin during the production process.

***
Conclusion:*** Hence, it seems; rifampin loaded inhalable proliposomes may be a suitable systemfor delivering liposomal rifampin into the lungs.

## Introduction

Tuberculosis (TB) is *Mycobacterium tuberculosis* induced debilitating infectious disease which caused 1.8 million deaths in 2015^1^ and is recognized as the second factor of mortality and morbidity in acquired immune deficiency syndrome (AIDS) patients.^2^ Therefore, it seems that the treatment and eradication of this life-threatening infection are very important. Pulmonary TB which involves the lungs, contributes more than 80% of TB pathology reported cases. Different approaches in global eradication of TB infection are achieving, but due to the slow production process and approval of new anti-TB drugs, there are a few new drugs close to reaching the market.^3^ Therefore, the currently recommended TB treatment regimen contains the classical combination of first-line drugs, including oral and parenteral administration isoniazid, rifampin, ethambutol and pyrazinamide which indicated more than 90% cure rates in drug-susceptible cases.^4,5^ However, this regimen is associated to some disadvantages, including poor bioavailability and high rates of the first-pass metabolism. Besides, long treatment duration (6 to 24 months) associated to unwanted side effects for patients, not only leads to low patient compliance and therapeutic failure^6-8^ but also it may emerge the new bacterial resistance.^9-11^ As a result, the new inhalable formulation design for available conventional drugs may be an effective strategy to overcome mentioned obstacles of TB treatment since it can localize the drug in the lung, which is the site of action, in addition, can decrease drug administered dose and systemic side effects while it may accelerate drugs’ onset of action.^7,12^ Among available three inhalation delivery systems, dry powder inhalers (DPIs) were rapidly improved due to their propellant-free nature with higher stability and dose capacity which leads to higher patients’ compliance.^8,13^ During past decades, many investigations have focused on three main categories of inhalable formulations for anti-TB drugs to enhance localized drug targeting, which include liposomes, microparticles and nanoparticles.^12,14^ Liposomes are lipid-containing vesicles formulated with exogenous phospholipids similar to lung surfactant that represent a promising drug carrier for pulmonary delivery.^1,12^ This carrier is stored in mononuclear phagocytic system cells, mainly macrophages where the *Mycobacterium tuberculosis* resides. Therefore, the carrier improves the effectiveness of anti-TB drugs.^4,15^ Besides, liposomes offer other advantages such as reduction of drug toxicity and side effects, high encapsulation efficacy, controlled drug release over a long period, and drug protection in destructive conditions, such as pH and enzyme.^16,17^ Despite liposomal benefits, these carriers have serious limitations like phospholipid oxidation, aggregation, sedimentation, instability and drug leakage. To eliminate the mentioned drawbacks, proliposomes were introduced, which were free-flowing powders that contained a hydrophilic carrier that was layered with phospholipids and form liposomal dispersion on hydration. The solid-state of proliposomes ensures chemical and physical stability, and improves the shelf-life of encapsulated drugs.^18-20^ Proliposomes production is a simple and reproducible manufacturing technique for large-scale production of liposomes.^19^ Proliposome carriers are water-soluble materials such as mannitol, sorbitol, maltodextrin, etc. Carriers with higher surface area and porosity may regulate the lipid amount required to make proliposomes.^19,21^ Proliposomes’ ingredients are very similar to mammalian membrane structures that result in higher biocompatibility and biodegradability and lead to suitable properties as pulmonary delivery of drugs.^20^ Proliposomes as DPIs are beneficial for pulmonary administration considering their reduced toxicity, enhanced potency, controlled drug delivery ability, and uniform lung deposition.^13^ Different inhalable proliposomes as the pulmonary carrier of different drugs including budesonide,^22^ ketotifen,^23^ amikacin,^24^ dapsone^13^ and levofloxacin^25^ were developed in previous studies. These proliposomes prepared using different phospholipids (hydrogenated soya phosphatidylcholine, egg phosphatidyl choline, dipalmitoyl phosphatidylcholine, cholesterol) and carriers (sucrose, lactose, mannitol), have shown proper aerosolization properties with successful *in vitro* lung deposition.

One of the most important parameters in aerosol delivery is aerodynamic diameter, which plays a key role in the deposition of particles within the respiratory system. Most of the dense powder aerosols indicate a density of 1 ± 0.5 g/cm^3^; however, porous particles with lower density (< 0.4 g/cm^3^) may deposit in lung deeper^26^ Considering the direct proportional of the aerodynamic diameter with the square root of particle density, the lower particle density leads to the smaller aerodynamic diameter. Porous carriers show low density with improved aerodynamic properties.^27^

The objective of this study was first to produce porous carriers using different porogen agents and then design and evaluate carrier porosity effect on inhalable rifampin-loaded proliposomes properties.

## Materials and Methods

### Materials

Rifampin was purchased from Hakim pharmaceutical company, Iran. Lactose, sucrose, mannitol, cholesterol, and acetic acid were obtained from Merck Chemicals Co., Germany. L-α-lecithin ((3-*sn*-phosphatidylcholine) from Soybean, Type IV-S, ≥30 enzymatic) was obtained from Sigma, USA. All the other chemicals and reagents were of analytical grade.

### Rifampin analysis validation

UV-vis spectrophotometer (T80, Germany) was applied at a maximum absorbance wavelength for rifampin quantification. Two analytical curves were plotted in water: ethanol (30:70) and phosphate buffer solution pH 7.4 as medium solutions. All rifampin concentrations were prepared on three different days, and every concentration was tested three times a day. The curves were validated by linearity, inter-day, and intra-day precision, and accuracy.

### Porous carrier preparation

To produce porous carrier, lactose (L) and mannitol (M) aqueous solutions (10% w/v) were used as main carriers, and sucrose and citric acid (1 and 2% w/v) were used as templating agents to induce porosity.^28^ Eight prepared solutions (L1-L4 and M1-M4) were spray dried using Dorsa spray drier, Iran, with pump rate 50%, aspirator 90 %, and inlet temperature 100 °C. The resultant powders were dispersed in ethanol to remove the templating agents. Carrier formulations are reported in Table 1.

**Table 1:** Selected experiments by software for proliposome formulations

	**F1**	**F2**	**F3**	**F4**	**F5**	**F6**	**F7**	**F8**	**F9**	**F10**	**F11**	**F12**	**F13**	**F14**	**F15**	**F16**	**F17**	**F18**	**F19**	**F20**
Rifampin (mg)	4.9	30.1	17.5	25	17.5	25	17.5	25	17.5	10	25	17.5	17.5	10	10	17.5	17.5	10	17.5	17.5
Lipid (mM)	350	350	350	500	350	200	350	500	97.7	500	200	350	350	200	500	602.3	350	200	350	350
Carrier (mg)	575	575	575	250	575	900	575	900	575	900	250	575	575	250	250	575	28.4	900	1121.6	575
Carrier/rifampin	117	19	33	10	33	36	33	36	33	90	10	33	33	25	25	33	2	90	64	33
Lipid/rifampin	71	12	20	20	20	8	20	20	6	50	8	20	20	20	50	34	20	20	20	20
Carrier/lipid	1.6	1.6	1.6	0.5	1.6	4.5	1.6	1.8	5.9	1.8	1.3	1.6	1.6	1.3	0.5	1.0	0.1	4.5	3.2	1.6

### Porous carrier characterization

#### Surface area

The surface area of powders was determined using ChemBet-3000, USA. Powders previously were degassed at 100°C for 3 hours and surface area values were reported as BET numbers. Non-porous lactose and mannitol were also analyzed as reference powders for the following comparisons.

#### Morphology

Selected porous powder morphology was analyzed using Scanning Emission Microscope (SEM) (Cambridge S-360, USA, 13Kv). Samples were spread on an aluminum stub, and then particles were golden sputtered. For proper comparison, the non-porous powder was also analyzed by this method.

### Proliposome preparation

Proliposomes were prepared using the slurry method. In brief, the lipid phase and rifampin were dissolved in chloroform and then mixed with carrier powder (in porous and non-porous forms). The obtained dispersion was transferred to the rotary evaporator (80 rpm, 40°C, IKA, Germany) to remove chloroform under vacuum. The final dry powder was passed through mesh 60 and maintained in a desiccator for complete drying. Rifampin-loaded proliposomes were optimized using quality by design method. Based on preliminary formulations, four factors of carrier type (porous and non-porous), carrier amount (200-900 mg), rifampin amount (10-25 mg), and lipid phase amount (200-500 mM) were selected as variables. Powder formulations were prepared by applying optimization design using Design Expert 10^®^ software (Stat-Ease, Inc.; Trial version). A randomized central composite response surface method was used for proliposome optimization. Different responses were analyzed, including proliposome powder weight yield, density, flowability and hydrated liposomes size, loading capacity and encapsulation efficiency. The software suggested 40 experiments (Table 1, 20 experiments for porous and 20 for non-porous carrier), including six center points to identify any curvature.

### Proliposome characterization

#### Weight yield

The yield was calculated by weighing the obtained powder divided by the theoretical weight. The weight yield was reported as a percentage, and the samples were examined in triplicate.

#### Flowability

Due to USP guidelines, a defined weight of proliposome powders was transferred to Erweka^®^Granule Flow tester, and their angle of repose was measured. All tests were repeated three times.

#### Bulk density

To determine the bulk density, a defined weight of the powders was transferred to a graduated cylinder. Then, the density of powders was calculated by the division of weight over the volume. Samples were tested in triplicate.

#### Drug content

Content uniformity was defined by measuring the total amount of rifampin in proliposomal particles. Definite amounts of proliposome formulations were dissolved in absolute ethanol until the lipid phase was dissolved completely. Then, the samples were centrifuged 30 minutes (4°C, 18 000 rpm). The supernatant rifampin content was analyzed using the validated analysis method. The process was repeated 3 times.

### Hydrated liposomes characterization

Hydrated liposomes were obtained by adding water to proliposomes. Typical characteristics of hydrated liposomes, including size, loading capacity and encapsulation efficiency were evaluated.

#### Size

The hydrated liposome size was analyzed using particle size analyzer (Shimadzu, SALD-2101, Japan). Mean volume and number diameters of liposomes were determined.

#### Loading capacity and encapsulation efficiency

A certain amount of proliposome was dispersed in distilled water and was shaken to obtain a colloidal dispersion, followed by centrifugation for 30 minutes at 18 000 rpm, 4°C. The supernatant was analyzed using the analysis method to quantify the amount of rifampin. The absorbance demonstrates the un-encapsulated drug content. Hence, the amount of trapped drug was calculated by subtracting the un-encapsulated value from the total amount of rifampin added to the formulation. Loading capacity is the amount of drug which was encapsulated in hydrated liposomes. Loading capacity (DL) and encapsulation efficiency (EE) were assessed using the following equations^29^:


$$Loading\;capacity\;\%=\frac{Encapsulated\;drug\left(mg\right)}{\mathrm{Pr}oliposome\;powder\;weight\left(mg\right)}\times 100$$

$$Encapsulatation\;efficiency\;\%=\frac{\mathrm{Pr}actical\;drug\;loading}{Theoretical\;drug\;loading}\times 100$$

### Final optimized proliposome characterization

Considering powder characterization for pulmonary delivery, powder density and flowability were the main parameters for optimization. In a more step, due to software results, the most significant effective parameter for hydrated liposome was extracted to determine the optimized formulation.

#### In vitro release

A known amount of final optimized proliposomes was dispersed in phosphate buffer solution pH 7.4 to create sink condition and incubated in a shaker incubator at 50 rpm for 8 hours at 37 ± 1°C. Samples were taken at 0.5, 1, 2, 4, 6, and 8 hours (n = 3) and analyzed using the analysis method described earlier.

#### In vitro aerosolization

Selected formulations were assessed for the aerosolization properties using a Next Generation Impactor (NGI, Copley Scientific, UK) at room temperature. The instrument was equipped with a USP induction port and pre-separator. Airflow of the instrument was retained at 60 L/min by a flow meter (DFM 2000, COPLEY scientific, UK). Prior to the study, all collection cups were coated using a solution of Tween 80 in ethanol (1% W/V). The optimized formulations were delivered to a size 3 capsule and were actuated to the NGI using an Aerolizer®. Finally, deposited drug concentration in each stage was determined using the validated UV-VIS analysis method. Mass median aerodynamic diameter (MMAD), geometric standard deviation (GSD), fine particle fraction (FPF), and powder recovery% were determined. All experiments were repeated three times.

#### Morphology

Morphology of selected proliposome formulations was evaluated by SEM (TESCAN-VEGA3 (Czech Republic), 10 kV). The selected samples were spread on an aluminum stub, and then particles were golden sputtered. Liposomes were formed by hydration in water, and their shape was examined by transmission electron microscope (TEM) (LEO 906E, Philips, Germanys). The sample was added on a formvar-coated grid and stained by 2% (w/w) uranyl acetate.

#### Differential scanning calorimetry (DSC)

DSC method (BAHR thermo analyzer, GmbH, Germany) was applied to study the solid-state and stability of rifampin in the proliposome particles. Rifampin, rifampin encapsulated proliposome and blank proliposome were set in aluminum pans and heated up to 350°C (heating rate of 10°C/min). The void aluminum pan was sealed as a reference sample.

### Statistical analysis

All experiments were done in triplicate (n = 3) and data were expressed as the mean ± standard deviations. Analysis of variance (ANOVA) via SPSS v.15 software was used for statistical analysis in which *P* value of <0.05 was considered to denote a statistically significant difference.

## Results and Discussion

### Rifampin analysis validation

Rifampin analysis was evaluated in water: ethanol (30:70) and phosphate buffer solution pH 7.4 at ƛ_max_ 475 nm. The data of curve validation containing the regression equation, the correlation coefficient (r^2^) of the standard curve, and the precise and accurate validation of the results of analytical curves are presented in Table 2. The validation results implied acceptable correlation coefficient, precision, and accuracy which approved the applied method for evaluation of rifampin in the rest of the study is valid.

**Table 2:** Validation parameters of different analytical curves of rifampin (n = 9)

**Solvent**	**Equation**	** r^2^**	**Precision% (Intraday)**	**Precision% (Interday)**	**Accuracy%**
Water: Ethanol (30:70)	y = 0.015x+0.009	0.999	99.6 ± 0.2	98.6 ± 1.2	98.2 ± 2.9
Phosphate buffer pH 7.4	y = 0.018x-0.014	0.999	98.4 ± 1.9	98.1 ± 1.4	97.7 ± 2.8

### Porous carrier characterization

#### Surface area

Porous particles with high void spaces and low density may promote aerosol powder performance.^30^ As previously reported, the templating method was used to prepare powder with higher porosity and surface area. Porous lactose and mannitol carriers were prepared by spray drying method using different templating agents, and the surface area of powders was determined and compared. As it is shown in Table 3, non-porous lactose and mannitol had surface areas of 0.30 ± 0.09 and 0.71 ± 0.1 m^2^/g, respectively. The highest area (24.95 m^2^/g) was determined for lactose samples containing sucrose 2% w/v as templating agent, which was 80 times higher than intact lactose, respectively. Similar results were reported in a previous study that examined different templating agents in the preparation of porous lactose carrier which indicated the highest BET number (20 ± 1 m^2^/g) for sucrose as a templating agent.^28^ Citric acid inefficiency in making porous carrier can be explained due to the Williams-Landel-Ferry theory which indicates that the lower glass transition temperatures (*T*g) of templating agent molecules leads to higher crystallization (aggregation) rates of templating agent during spray drying. Therefore, citric acid with glass transition temperatures of 11°C would be crystallized rapidly while sucrose acid with glass transition temperatures of 62°C crystallized slowly.^28,31^ However, citric acid pH effect on the degree of crystallinity in the powder is an important factor. Citric acid with low pH may increase lactose crystallization due to the increasing the mutarotation and orientation rate of lactose molecules into the crystals, which may form more aggregates.^32^ Due to BET results, it seems that lactose with 2% sucrose as the templating agent may be a more appropriate carrier for proliposomes production due to its higher surface area and porosity.

**Table 3:** Different spray dried carrier formulations surface area determined by BET test (m^2^/g) (n = 3)

	**Carrier (Lactose)**	**Templating agent % (w/v)**	**Surface area (m** ^ 2 ^ **/g)**		**Carrier (Mannitol)**	**Templating agent % (w/v)**	**Surface area (m** ^ 2 ^ **/g)**
L0	Non porous	-	0.30 ± 0.09	M0	Non porous	-	0.71 ± 0.1
L1	Porous	Sucrose 1	13.67 ± 0.95	M1	Porous	Sucrose 1	3.14 ± 0.14
L2	Porous	Sucrose 2	24.95 ± 1.13	M2	Porous	Sucrose 2	4.64 ± 0.30
L3	Porous	Citric acid 1	2.69 ± 0.34	M3	Porous	Citric acid 1	3.02 ± 0.11
L4	Porous	Citric acid 2	3.35 ± 0.23	M4	Porous	Citric acid 2	3.36 ± 0.57

#### Morphology

**Figure 1:**
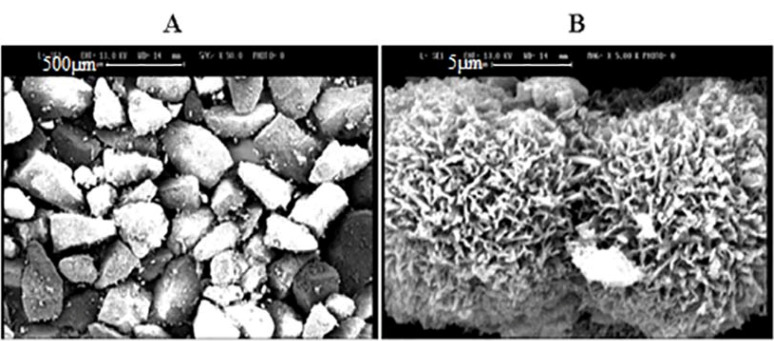
SEM graphs of powders from (A) lactose (scale bar 500µm) and (B) porous lactose (scale bar 5 µm).

Porous and non-porous powders morphologies were studied by SEM (Figure 1). As it is shown, non-porous powders (A) had smooth and un-textured surfaces with no assignable pore. In comparison, the porous powder had a high intensity of asperities on the surface (B) by the uniform distribution of pores; which can be ascribed to the proper dispersion of templating molecules in the lactose structure. The data is based on the BET data and approves the creation of a high surface area in lactose after ethanol washing.

### Proliposome preparation

Different methods were employed for proliposome production on a large scale, including spray drying and fluidized bed coating; however, traditionally feed-line method utilizing rotary evaporator is achieved for the small-scale process. Considering lipid losses in the feeding tube and long-lasting process, finding a simple alternative production method would be highly advantageous.^33^ Therefore, in the present study slurry method was used for proliposome production.

Based on the software, 40 formulations containing 20 formulations for porous and 20 formulations for non-porous carriers were designed and prepared. Powders weight yield, flowability and density were determined. The best-fitting models were selected based on the statistical parameters including lack of fit (shows the fitness of the model), the multiple correlation coefficients (R^2^, approves the correlation coefficient and reaches 1 as the results become better), predicted multiple correlation coefficients (predicted R^2^, measurement of the predictive capability of the model), and adjusted multiple correlation coefficients (adjusted R^2^, adjustment of the number of model parameters relative to the number of runs). The difference between predicted R^2^ and adjusted R^2^ values should be less than 0.2 for the proper prediction of the model.

### Proliposome characterization

#### Weight yield

Weight yield for the prepared proliposome is shown in Table 4. Non-porous lactose powders weight yield was in a lower range of 47- 78%, and just NP18 showed 83%, while porous lactose powders weight yield was in a higher range of 53-85%. Formulations P17 and NP17 had the lowest weight yield, which would be due to the lowest carrier/lipid and carrier/rifampin ratio that caused stickiness to the rotary evaporator flask. Therefore, it was omitted for further evaluation.

**Table 4:** Characterization of proliposome powders

	**Weight yield (%)**	**Angle of repose**	**Density**		**Weight Yield (%)**	**Angle of repose**	**Density**
P1	77 ± 3.1	19.6 ± 0.3	0.35 ± 0.03	NP1	71 ± 2.2	23 ± 0.7	0.51 ± 0.03
P2	65 ± 2.9	22.1 ± 0.6	0.34 ± 0.02	NP2	65 ± 3.1	24.4 ± 0.8	0.52 ± 0.04
P3	72 ± 2.3	16.2 ± 0.4	0.43 ± 0.01	NP3	66 ± 2.1	23 ± 0.3	0.54 ± 0.01
P4	53 ± 2.1	40.3 ± 1.0	0.36 ± 0.02	NP4	52 ± 1.4	22.5 ± 0.5	0.47 ± 0.03
P5	71 ± 4.1	16.0 ± 0.3	0.40 ± 0.03	NP5	67 ± 2.8	23.2 ± 0.2	0.53 ± 0.02
P6	82 ± 6.0	15.3 ± 1.0	0.40 ± 0.01	NP6	76 ± 3.8	22.8 ± 0.6	0.55 ± 0.02
P7	70 ± 5.4	15.8 ± 0.9	0.42 ± 0.02	NP7	64 ± 4.1	22.8 ± 0.6	0.53 ± 0.01
P8	78 ± 6.3	14.5 ± 0.8	0.38 ± 0.03	NP8	70 ± 3.6	24.5 ± 0.3	0.52 ± 0.01
P9	79 ± 2.4	20.4 ± 0.4	0.31 ± 0.03	NP9	67 ± 3.4	19.7 ± 0.7	0.54 ± 0.01
P10	85 ± 3.2	12.5 ± 0.9	0.42 ± 0.01	NP10	71 ± 2.5	22.7 ± 0.3	0.47 ± 0.01
P11	66 ± 1.8	25.0 ± 0.5	0.36 ± 0.03	NP11	54 ± 2.3	25.5 ± 0.3	0.46 ± 0.02
P12	70 ± 1.4	15.5 ± 0.5	0.42 ± 0.02	NP12	61 ± 3.9	22.1 ± 0.3	0.52 ± 0.03
P13	71 ± 3.5	13.5 ± 0.7	0.40 ± 0.03	NP13	60 ± 3.1	22.5 ± 0.5	0.54 ± 0.03
P14	72 ± 2.7	19.8 ± 0.3	0.29 ± 0.02	NP14	59 ± 1.9	27.9 ± 0.4	0.46 ± 0.01
P15	63 ± 3.1	30 ± 0.7	0.45 ± 0.03	NP15	47 ± 2.2	23.5 ± 0.5	0.46 ± 0.02
P16	71 ± 3.8	21.2 ± 0.7	0.38 ± 0.01	NP16	60 ± 2.7	20.6 ± 0.7	0.51 ± 0.03
P17	-	-	-	NP17	-	-	-
P18	85 ± 2.9	11.9 ± 0.4	0.27 ± 0.03	NP18	83 ± 2.4	24.3 ± 0.3	0.53 ± 0.02
P19	77 ± 4.1	16.0 ± 0.6	0.40 ± 0.02	NP19	78 ± 3.6	22.9 ± 0.5	0.47 ± 0.03
P20	66 ± 1.5	19.6 ± 0.5	0.42 ± 0.04	NP20	64 ± 2.7	23.0 ± 0.7	0.54 ± 0.01

(P: porous, NP: non-porous).

#### Flowability 

The flowability of powders (Table 4) was evaluated by measuring the angle of repose and comparing it with mentioned USP indexes. The angle of repose describes the flow characteristics and powder friction. The small angle of repose (< 30) is an indication of lower powder internal friction and cohesiveness.^34^ The angle of repose for non-porous formulations showed a narrower range 19.7-27.9, compared to porous formulations (12.5-40) with a wider range. All porous formulations’ angle of repose was in the acceptable range except P4 and P15 with higher amounts which may be related to the lowest carrier/lipid ratio, which contains the highest lipid with the lowest carrier amount in the formulation. The excellent flow properties were obtained when the angle of repose was between 25 to 30, and it was fair flowability property when the range was between 36 to 40.

Due to the software, the lack of fit of this criterion was not significant. R-squared was 0.97, and the difference between Pred R^2^ and Adj R^2^ was less than 0.2. Adeq precision of 22.06 indicates an adequate signal.

**Angle of repose** = +31.57278-0.8594*Drug-0.018695*Lipid-9.00735E-003*Carrier +8.96667E-004*Drug*Lipid-7.42051E004*Drug*Carrier-7.72564E-005*Lipid*Carrier +0.032838*Drug^2^+8.14485E-005*Lipid^2^+2.47549E-005*Carrier^2^

#### Bulk density

The bulk density of prepared powders is mentioned in Table 4. The density of porous formulations was in the range of 0.27 to 0.45 g/mL, while in non- porous formulations, this factor was in the range of 0.46 to 0.56 g/mL; therefore, as it was expected, the porous particles with higher surface area showed lower density. P18 with the higher carrier/lipid and carrier/rifampin ratio had the lowest bulk density. Due to the previous studies, particles with a density lower than 0.4 g/ mL could enter the lower parts of the respiratory tract containing alveoli and be more effective in therapeutic regimens.^26^ Due to the software, the lack of fit of this criterion was not significant. R-squared was 0.97 and the difference between Pred R^2^ and Adj R^2^ was less than 0.2. Adeq precision of 19.56 indicates an adequate signal. Based on the mentioned results, non- porous formulations were eliminated, and formulations with porous carriers were examined in further assays.

**Density** = -0.088085 +0.025227*Drug +1.65059E-003*Lipid -8.16685E005*Carrier -3.33333E-005*Drug*Lipid+7.17949E-006*Drug*Carrier+1.00176E021*Lipid*Carrier-4.97979E004*Drug^2^-1.26136E006*Lipid^2^ -4.91113E-008*Carrier^2^

#### Drug content

As it was mentioned, porous proliposomes were selected for further assays. The drug content of the prepared formulations is mentioned in Table 5. The indirect method was applied to estimate the amount of rifampin in proliposome powders. All samples were in the acceptable range.

**Table 5:** Characterization of hydrated liposome vesicles

	**Drug Content (%)**	**Volume diameter (μm)**	**Encapsulation efficacy (%)**	**Loading capacity (%)**
P1	93.9 ± 0.32	39.50 ± 0.5	72.3 ± 2.6	1.40 ± 0.03
P2	97.4 ± 0.21	7.21 ± 0.55	34.1 ± 3.4	4.90 ± 0.41
P3	105.4 ± 0.45	4.04 ± 0.48	53.0 ± 1.6	3.30 ± 0.28
P4	100.6 ± 0.76	4.27 ± 0.50	52.0 ± 3.9	6.31 ± 0.19
P5	105.4 ± 0.89	4.10 ± 0.67	43 ± 2.3	2.80 ± 0.14
P6	103.5 ± 0.31	11.81 ± 0.50	26 ± 1.5	5.80 ± 0.30
P7	101.0 ± 0.65	4.85 ± 0.64	53 ± 2.1	3.90 ± 0.51
P8	106.5 ± 0.74	3.85 ± 0.47	39.8 ± 4.7	3.10 ± 0.12
P9	101.0 ± 0.49	10.78 ± 0.54	28.5 ± 1.9	6.40 ± 0.43
P10	106.5 ± 0.44	11.78 ± 0.60	58.5 ± 3.6	1.71 ± 0.07
P11	110.7 ± 0.39	4.21 ± 0.56	47.6 ± 2.3	6.91 ± 0.34
P12	101.1 ± 0.26	4.83 ± 0.72	54.7 ± 1.7	2.91 ± 0.19
P13	101.0 ± 0.12	4.16 ± 0.34	48.7 ± 2.1	3.04 ± 0.27
P14	103.1 ± 0.65	3.93 ± 0.51	81.8 ± 3.1	4.53 ± 0.32
P15	86.0 ± 0.90	3.21 ± 0.49	99.3 ± 3.3	2.70 ± 0.15
P16	104.6 ± 0.12	3.41 ± 0.47	78.8 ± 2.6	3.24 ± 0.25
P18	113.1 ± 0.43	7.89 ± 0.56	60.3 ± 2.2	3.70 ± 0.19
P19	101.0 ± 0.91	19.47 ± 0.75	28.3 ± 3.8	2.41 ± 0.36
P20	93.9 ± 0.67	4.84 ± 0.56	50.7 ± 1.6	3.22 ± 0.32

### Hydrated liposome characterization

#### Size

The mean volume diameter of hydrated liposomal vesicles was in the range of 3.21 to 7.8 μm except for P1, P10 (Table 5), which contain higher lipid/drug ratio and P6, P9 with the highest carrier/lipid ratio. Due to the software modeling, this criterion was not significant in the optimization of formulations. As reported in previous studies, the slurry method resulted in smaller particles size and variability in comparison with other methods. Besides, hydrated liposomes with 4-5.5 μm size range showed proper inhalation properties in volunteers that inhaled the liposomes by jet nebulizers.^30,33^ Results indicated a similar size of 3-4 micron for all formulations except P2, P18 (7-8 micron), P1, P6, P9, P10, and P19 (11-39.5 micron), which may attribute to higher carrier/lipid, carrier/rifampin and lipid/ rifampin ratios of mentioned formulations.^30^

#### Loading capacity and encapsulation efficiency

Liposomal encapsulation efficacy was between 21.5, and 78% and rifampin loading was 1.4 to 6.9% in different formulations (Table 5). Previous reports indicated higher encapsulation efficiency for liposomes prepared using the slurry method.^33^ P4, P9, and P11 had the highest amount of rifampin loading that would be due to the highest ratio of drug to lipid. The results indicated that a reduction in the lipid/carrier ratio that indicated a negative effect on encapsulation efficiency, especially in the cases of P6 and P9, while increasing this ratio in P4, P14, P15, and P16 led to positive effects on encapsulation efficiency. Lack of fit for loading capacity criteria was not significant. R-squared values were 0.97 for loading capacity, and the difference between Adj and Pred R^2^ was less than 0.2. Adeq precision of 19.49 for loading capacity indicates an adequate signal

**Loading capacity** = +7.00687+0.16764*Drug- 0.024048*Lipid-1.86723E-003* Carrier+6.32413E-005*Drug*Lipid-1.28377E-004*Drug*Carrier-6.03694E-006*Lipid*Carrier+9.74574E-004*Drug^2^+2.88901E-005*Lipid^2^+3.15089E-006*Carrier^2^

### Final optimized proliposome characterization

**Figure 2:**
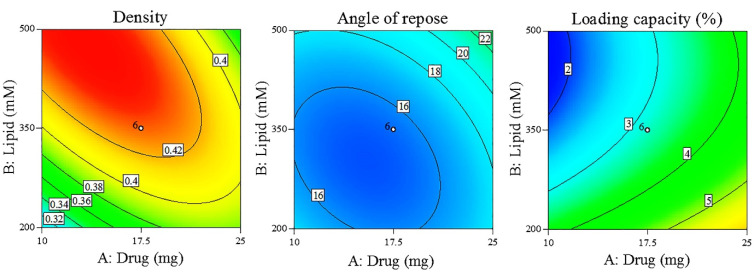
The contour plots of density, angle of repose and loading capacity.

The selection of final formulations, prepared with the porous carriers, was based on the obtained results in terms of proliposome density, flowability, and hydrated liposomes loading capacity (Table 6). Using the lowest powder density in the software, it proposed P18 as the optimized formulation (Figure 2). P18 was selected formulation considering density and flowability (angle of repose) as the main factors in combination. In the next step, in addition to powder density and flowability, liposomes loading capacity was also proposed as another main factor and the overlay counterplot as illustrated in Figure 2 showed P11 as the best formulation. Density was selected as the main factor in proliposome characteristics since density can affect powder aerosolization characteristics.^35^ Therefore, P11 and P18 formulations were evaluated for further controls.

**Table 6:** Factorial experimental design analysis of variance obtained

	**Angle of repose**	**Density**	**Drug loading**
R-squared	0.9713	0.9890	0.9672
Adj R-squared	0.9426	0.9774	0.9344
Pred R-squared	0.7629	0.9216	0.7670

#### In vitro release

**Figure 3:**
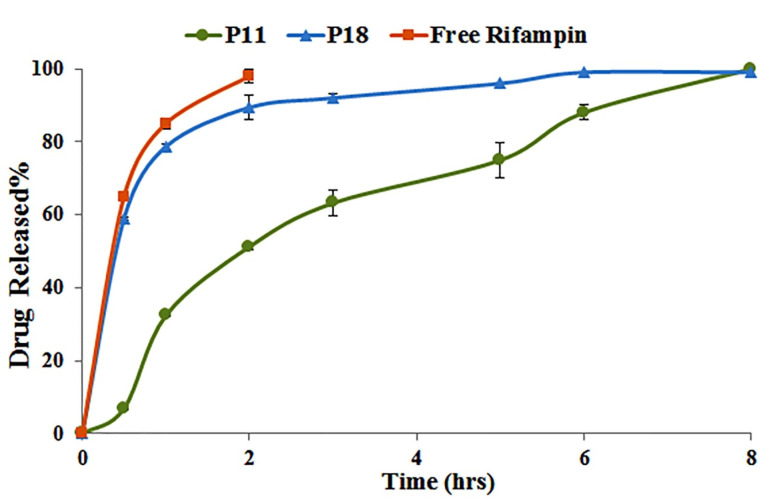
*In vitro* release profile of selected formulations in phosphate buffer solution pH 7.4 (n = 3).

As presented in Figure 3, 85% of Free rifampin was dissolved in release media within 1 hour and it was completely dissolved after 2 hours while it was last 6 hours for both optimized formulations to release rifampin; however, P18 showed the faster rifampin release compared to P11. Rifampin release rate was 8.5, 2.5, and 1.7 times higher in P18 after 0.5, 1, and 2 hours, which could be attributed to high carrier/rifampin, lipid/rifampin, and carrier/lipid in P18. Higher ratios of the carrier increase the probability of rifampin entrance into the carrier pores that could lead to the burst release^30^ of rifampin that is shown in P18 formulation.

#### In vitro aerosolization

**Figure 4:**
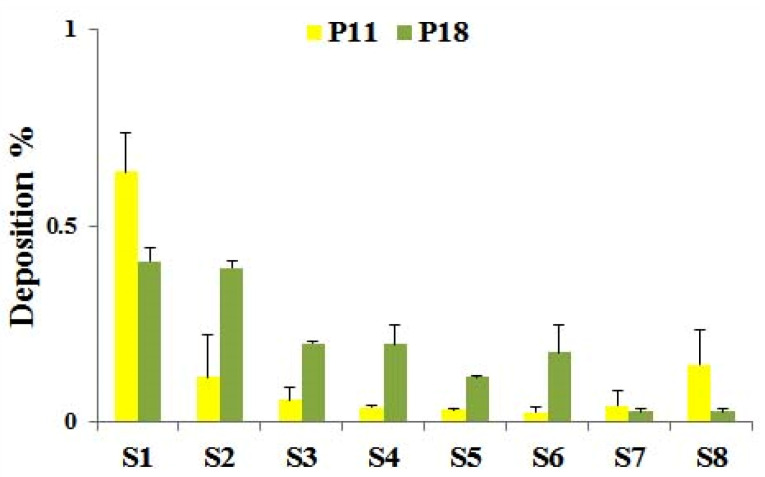
Deposition patterns of optimized proliposome powder in the NGI.

NGI evaluated inhalation properties of the proliposome powders are mentioned in Table 7. NGI deposition pattern presented in Figure 4. The aerodynamic diameter of P11 and P18 was in the acceptable range (1-10 μm), while P18 showed a little larger size. GSD was in an acceptable range for P18 formulation that was less than 2.5.^36^ Both formulations had high sedimentation in the upper parts of the NGI. This was predictable since the lipid nature of proliposomes would enhance the sedimentation rate. These results were approximately similar to previous reports that showed almost low FPF% of 15% for non-porous and 19-29% for the porous carriers.^25,27^ Moreover, another study reported low FPF (0 to 3.99%) for proliposomes with lactose as the carrier.^30^ The low FPF% for proliposomes may be related to the agglomeration of particles that were coated with lipid superficially.^27^ Higher FPF% and powder recovery% of P18 may related to the higher carrier/lipid and carrier/rifampin ratio in the formulation.^30^ Due to the obtained results, P18 was selected as the final formulation with the best powder characteristics.

**Table 7:** Optimized proliposomes *in vitro* aerosol assessment

	**P11**	**P18**
MMAD	5.25 ± 0.02	6.21 ± 0.36
GSD	5.42 ± 0.80	2.36 ± 0.25
FPF%	0.71 ± 0.06	9.17 ± 0.18
Emitted dose (mg)	0.95 ± 0.21	1.63 ± 0.25
Powder recovery%	75.91 ± 3.52	90.15 ± 3.01

#### Morphology

**Figure 5:**
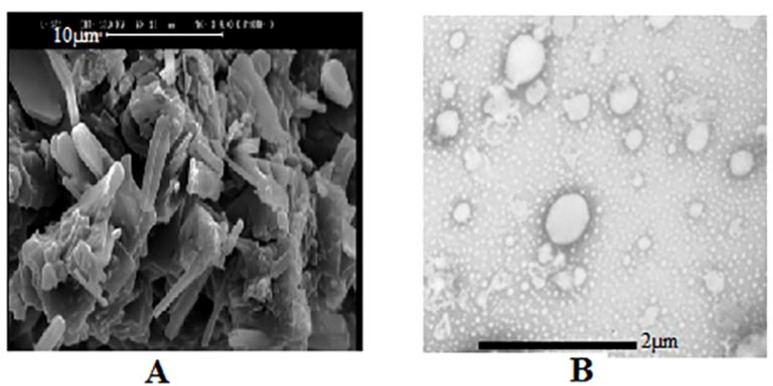
(A) SEM of proliposome P18 (scale bar 10µm), (B) TEM of hydrated liposomes of P18 (scale bar 2 µm).

SEM evaluated P18 formulation morphology. To confirm a liposome formation in the next step, the formulation was in contact with water to form liposomal vesicle and was evaluated by TEM. As it is presented in Figure 5, proliposome powders have a linear structure that could form into oily droplets in a liposomal vesicle state. The comparison of proliposome (P18) and porous lactose structure in Figure 1 showed the lipid phase clearly. Due to previous reports, decreasing the carrier ratio to less than 80% would form linear sticky particles.^27^ TEM picture in Figure 5 showed liposomal vesicles as oily droplets, which confirmed liposome formation.

#### Differential scanning calorimetry 

**Figure 6:**
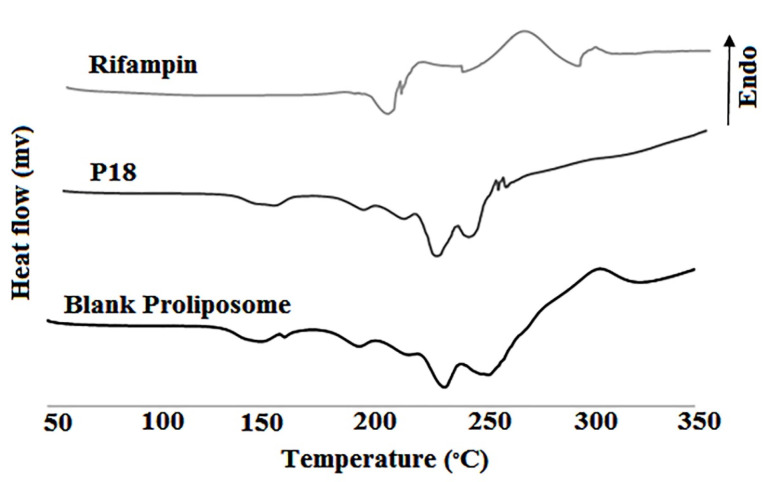
DSC thermograms of rifampin, blank proliposome and P18 formulation

Thermograms of rifampin, blank proliposome and P18 proliposome are in Figure 6. Rifampin is a polymorphic compound that showed the endothermic peak of melting point (195.2°C) and the exothermic peak (211.6°C) that is related to the recrystallization and converting to type I polymorph. The exothermic peak at 258.5°C is related to the destruction of rifampin.^37,38^ In the P18 formulation thermogram, rifampin exothermic peak at 225°C is obvious that confirms the presence of intact rifampin in proliposome powder.

## Conclusion

The effect of different factors such as carrier type, lipid phase amount and drug amount was evaluated on the powder characteristics of rifampin inhalable proliposome powder. The results of present study indicated that sucrose could enhance the porosity of lactose and its surface area. Besides, proliposome preparation by the slurry method was a proper method in laboratory scale to load rifampin in liposomes without destruction. The selected formulation showed almost acceptable *in vitro* aerosolization properties such as MMAD, GSD and emitted dose while FPF% was low which was related to the lipid nature of proliposome powder. Considering the optimized formulation suitable properties further studies with other different lipids may be needed to improve FPF% of rifampin-loaded proliposomes. Rifampin-loaded proliposomes with porous carriers showed acceptable aerosolization properties comparing non-porous carriers. Therefore, carrier porosity was an important parameter specially in aerosolization properties.

## Funding

This study was part of the Pharm.D project of Delaram Sadeghinia and was financially supported by Shiraz University of Medical Sciences.

## Ethical Issues

Not applicable.

## Conflict of Interest

Authors declare no conflict of interest.
